# Systematic Analysis of Environmental Chemicals That Dysregulate Critical Period Plasticity-Related Gene Expression Reveals Common Pathways That Mimic Immune Response to Pathogen

**DOI:** 10.1155/2020/1673897

**Published:** 2020-05-05

**Authors:** Milo R. Smith, Priscilla Yevoo, Masato Sadahiro, Ben Readhead, Brian Kidd, Joel T. Dudley, Hirofumi Morishita

**Affiliations:** ^1^Department of Psychiatry, Icahn School of Medicine at Mount Sinai, 1 Gustave L Levy Place, New York NY 10029, USA; ^2^Department of Genetics and Genomic Sciences, Icahn School of Medicine at Mount Sinai, 1 Gustave L Levy Place, New York NY 10029, USA; ^3^Nash Family Department of Neuroscience, Icahn School of Medicine at Mount Sinai, 1 Gustave L Levy Place, New York NY 10029, USA; ^4^Department of Ophthalmology, Icahn School of Medicine at Mount Sinai, 1 Gustave L Levy Place, New York NY 10029, USA; ^5^Institute for Next Generation Healthcare, Icahn School of Medicine at Mount Sinai, 1 Gustave L Levy Place, New York NY 10029, USA; ^6^Friedman Brain Institute, Icahn School of Medicine at Mount Sinai, 1 Gustave L Levy Place, New York NY 10029, USA; ^7^Mindich Child Health & Development Institute, Icahn School of Medicine at Mount Sinai, 1 Gustave L Levy Place, New York NY 10029, USA; ^8^ASU-Banner Neurodegenerative Disease Research Center, Biodesign Institute, Building A, 1001 S McAllister Ave, Tempe, AZ 85281, USA

## Abstract

The tens of thousands of industrial and synthetic chemicals released into the environment have an unknown but potentially significant capacity to interfere with neurodevelopment. Consequently, there is an urgent need for systematic approaches that can identify disruptive chemicals. Little is known about the impact of environmental chemicals on critical periods of developmental neuroplasticity, in large part, due to the challenge of screening thousands of chemicals. Using an integrative bioinformatics approach, we systematically scanned 2001 environmental chemicals and identified 50 chemicals that consistently dysregulate two transcriptional signatures of critical period plasticity. These chemicals included pesticides (e.g., pyridaben), antimicrobials (e.g., bacitracin), metals (e.g., mercury), anesthetics (e.g., halothane), and other chemicals and mixtures (e.g., vehicle emissions). Application of a chemogenomic enrichment analysis and hierarchical clustering across these diverse chemicals identified two clusters of chemicals with one that mimicked an immune response to pathogen, implicating inflammatory pathways and microglia as a common chemically induced neuropathological process. Thus, we established an integrative bioinformatics approach to systematically scan thousands of environmental chemicals for their ability to dysregulate molecular signatures relevant to critical periods of development.

## 1. Introduction

Millions of newly synthesized chemical substances are added to the global inventory each year [1]. Tens of thousands of these are commercially produced and may be exposed to human beings [2]. Our dedication to generating this impressive chemical inventory has not been matched by our capacity to screen these chemicals for their impact on human brain development. Neurodevelopmental disorders are highly prevalent, occurring in 17% of children, and this rate may be increasing [3], demanding serious consideration of how synthetic chemicals introduced into the human environment impact brain development. Human and animal studies have demonstrated that a number of environmental chemicals profoundly disrupt prenatal neural events such as proliferation, migration, and differentiation, leading to severe neurodevelopmental disorder [4]. In contrast, identification of chemicals impacting postnatal and childhood neurodevelopment has received less effort.

During childhood, the human brain undergoes refinement and reorganization during windows of heightened brain plasticity. These critical periods allow refinement of brain circuits by sensory and social experiences, which helps to establish normal perception and higher cognitive function [5–10]. Disruption of these critical periods can alter neural circuits that shape function and behavior, which may in turn contribute to neurodevelopmental disorders such as autism [11, 12].

Despite the potential for deleterious impacts on health, the role of environmental chemicals on critical period neuroplasticity has received minimal attention, although a few disruptors of developmental plasticity have been identified, including alcohol and bisphenol A [13, 14]. However, given the number of synthetic chemicals present in the environment, we need systematic approaches in order to accelerate the discovery of chemicals that damage brain development.

In our proof-of-principle study, we applied an integrative bioinformatics approach to assess hundreds of known neurotoxicants; using this strategy, we were able to rapidly identify and demonstrate that lead (Pb) disrupts critical period brain plasticity [15]. In this study, we built on that proof-of-principle, scanning across thousands of environmental chemicals to identify those that dysregulate two gene signatures of visual cortex critical period plasticity in mice. Among the 50 chemicals that dysregulated both gene signatures, we identified enrichments of common immune pathways, implicating microglia and inflammatory pathways in the pathology induced by exposure to these chemicals. Our findings show that an integrative bioinformatics approach is well suited to systematically assess the vast chemical space to identify candidate compounds that disrupt brain development.

## 2. Methods

### 2.1. Critical Period Plasticity-Related Signatures

Critical period signatures were generated from publicly available data obtained from juvenile and Lynx1-/- mice ([16]; GSE89757). Briefly, transcriptomes from the primary visual cortex (V1) in juvenile C57BL/6 mice on postnatal day (P) 29, adult Lynx1-/- mice (>P60), and adult C57BL/6 (>P60) mice (*n* = 3 each group) were profiled by microarray. Probe-level data were background corrected, quantile-normalized, and log2-transformed with Limma [17], yielding 9657 genes that mapped to human orthologues according to the Mouse Genome Informatics homology reference. Critical period signatures were defined as differential gene expression across the 9657-gene transcriptome in juvenile wild-type or Lynx1-/- adult vs. wild-type adult.

### 2.2. Environmental Chemical Signatures

Chemical signatures were derived as gene sets from Comparative Toxicogenomics Database (CTD) data. Only the chemical-mRNA relationships but not the chemical-protein relationships were extracted from 1.25 million CTD relationships between chemicals and 33 biological substrates (protein, DNA, mRNA, etc.). We only kept the chemical-mRNA relationships associated with PubMed references. To maximize power to detect biological and chemical characteristics in downstream analysis, all chemicals, including biologics and chemicals with unknown relevance to human exposure, were retained. Three gene set libraries consisting of groups of genes differentially expressed by a given chemical were created, limiting gene members to those also expressed in the critical period transcriptomes consisting of the 9657 genes after filtering for a minimum gene number filter of 3 genes: (1) CHEM composite (2001 chemicals; 3–750 genes per gene set), consisting of genes whose expression was either increased or decreased by a given chemical; (2) CHEM up (1742 chemicals; 3–726 genes per gene set), consisting of genes that were increased by a given chemical; and (3) CHEM down (1242 chemicals; 3–653 genes per gene set), consisting of genes that are decreased by a given chemical. Note that there are overlaps of chemicals among three libraries as CHEM composite gene sets were split into CHEM up and CHEM down libraries.

### 2.3. Molecular Matching

Gene Set Enrichment Analysis (GSEA) was used to assess the transcriptional similarity between a given chemical and the critical period signatures. GSEA was selected over other methods, such as the Connectivity Map approach [18], because GSEA controls the size of the input gene set (e.g., chemical gene sets) in its false discovery rate (FDR) calculation, which otherwise generally correlates with a *P* value; this is ideal in this context given the wide range of our chemical signature sizes (3 to 750 genes). Molecular matches using GSEA were computed between the CHEM composite, CHEM up, and CHEM down libraries and the juvenile and Lynx1-/- signatures; matches were considered significant if *P* < 0.05 and FDR < 0.25. An FDR of 0.25 was chosen for this exploratory discovery study to find candidate hypothesis to be further validated as a result of future research while avoiding overlooking potentially significant results. An initial exploratory GSEA was performed to assess whether CHEM composite signatures tended to impact expression of genes up- or downregulated in the juvenile and Lynx1-/- critical period signatures, as determined by the binomial test. Given that genes belonging to the CHEM composite signatures were much more likely to yield negative GSEA scores, indicating that they were among the downregulated genes in both juvenile and Lynx1-/- signatures, we then assessed separately if chemicals increased or decreased these genes applying GSEA to the 1742 CHEM up signatures and the 1242 CHEM down signatures.

### 2.4. Chemogenomic Enrichment Analysis

To uncover neurobiology of the 50 candidate plasticity-disrupting chemicals, we applied chemogenomic enrichment analysis (CGEA) to identify biological pathways overrepresented among the 50 chemicals relative to the remaining 1692 CHEM up signatures. To do so, we calculated gene set enrichment for 5191 Gene Ontology (GO) Biological Processes (BP) and for 96 Library of Integrated Network-based Cellular Signatures (LINCS) ligand expression profiles, using Fisher's exact test to assess the likelihood that genes overlapped between a given CHEM up signature and a given GO BP or ligand pathway. Enrichments were binarized to 1 if Padj < 0.05 and to 0 otherwise, and a hypergeometric test as implemented in the hypergea R package [19] was performed for each of 5191 GO BP and 96 LINCS ligand profiles to determine whether a given pathway was more likely to have a chance to be enriched in the 50 CHEM up signatures compared to the 1692 chemicals in the background.

### 2.5. Human Exposure Annotations

The risk of human exposure for a given chemical was determined from the literature, using the PubMed and Google Scholar search tools. Specifically, each name of the 50 chemicals derived from informatics analysis was searched in combination with other key terms such as “neurodevelopment”, “neurotoxin”, “neurotoxicity”, “neurological side effects”, and “cognitive development”. We added more explanation to this section in Discussion. We identified 11 chemicals as high exposure risk, 14 as medium exposure risk, and 25 as low exposure risk. For example, chemicals like pyridaben, which are commonly detected on agricultural produce consumed by humans [20], were considered a high risk for exposure. In contrast, tool chemicals that are only used in the laboratory, such as SB-431542, were considered low risk. Medium risk included chemicals such as medications that are no longer the primary prescription for a given indication.

### 2.6. Activated Microglia Gene Set Enrichment

A total of 72 genes that increased by lipopolysaccharide- (LPS-) activated microglia were identified from the supplementary tables of a previous study [21]. Enrichments between the activated microglia genes and each of the 50 CHEM up signatures were calculated using Fisher's exact test, using as a background 15071 genes expressed in both microglia and CTD chemicals.

### 2.7. Statistical Analyses

Statistical analyses were completed in the R programming language (v 3.2.2). In cases of multiple hypothesis testing, *P* values were corrected using the false discovery rate (FDR) approach [22]; the corrected values are referred to as *P* adjusted (Padj) throughout the manuscript.

## 3. Results

### 3.1. Molecular Matching of Critical Period and Environmental Chemical Signatures

**Figure 1:**
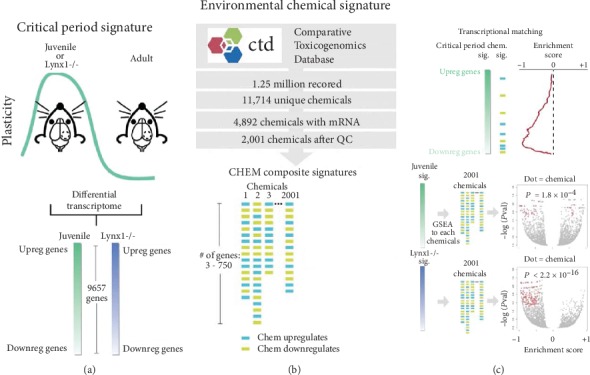
Environmental chemicals preferentially impact expression of genes downregulated in the critical period brain plasticity signatures of juvenile and Lynx1-/- mice. (a) We generated two in vivo critical period transcriptome signatures (juvenile at the peak of the endogenous critical period at P26 and Lynx1-/- adult mice, which maintain critical period-like plasticity) from public data. (b) Environmental chemical signatures using genes either increased or decreased by a given chemical (CHEM composite) were derived from the Comparative Toxicogenomics Database. (c) Molecular matches were computed to the critical period signatures using Gene Set Enrichment Analysis (GSEA) to identify that chemicals preferentially impact genes downregulated in the critical period signatures.

**Figure 2:**
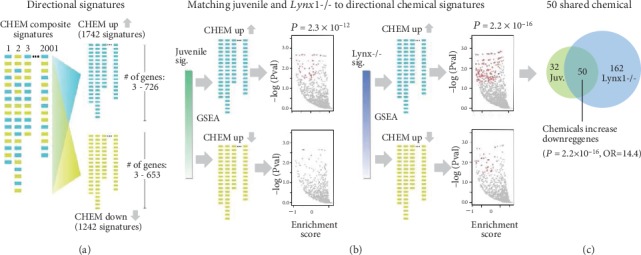
Molecular matching via GSEA identifies 50 chemicals that increase expression of genes downregulated in the juvenile and Lynx1-/- critical period transcriptome signatures. (a) 2001 CHEM composite gene sets were split into CHEM up (1742 signatures) and CHEM down (1242 signatures) libraries to assess the directional impact of each chemical on critical period gene expression. (b) GSEA was used to assess negative scores (reflecting a chemical's impact on downregulated critical period genes) for CHEM up and CHEM down signatures against the critical period signatures and the binomial test to assess a bias to up or down library. (c) Fifty chemicals increase downregulated critical period genes. See Supplementary Table 1 for a list of all 50 chemicals.

We generated two critical period signatures from transcriptomes of the primary visual cortex (V1) of juvenile wild-type mice during the peak of the critical period for visual cortex-mediated ocular dominance plasticity at postnatal day (P) 26 [23] or adult Lynx1-/- mice that have open-ended critical period plasticity throughout life [24] in comparison with adult wild type, revealing differential expression of 9657 genes (signatures derived from GSE89757 [16]) (Figure 1(a)). To determine the impact of environmental chemicals on juvenile and Lynx1-/- plasticity signatures, we used GSEA [25] to compute molecular matches of chemical gene expression signatures derived from the Comparative Toxicogenomics Database (CTD) to critical period signatures. Using 2001 composite chemical signatures (i.e., genes either increased or decreased by a given chemical, referred to as CHEM composite) (Figure 1(b)), we found that chemicals were more likely to impact the expression of genes that were downregulated in juvenile and Lynx1-/- critical period signatures, rather than genes that were upregulated (binomial tests: *P* = 1.8 × 10^−4^ and *P* < 2.2 × 10^−16^) (Figure 1(c)). Because environmental chemicals preferentially impact genes downregulated in the critical period signatures, we used GSEA to compute molecular matches between the directional chemical signatures (CHEM up: sets of genes increased by 1742 chemicals; CHEM down: sets of genes decreased by 1242 chemicals) and assessed only negative GSEA scores (reflecting a chemical's impact on downregulated critical period genes) to find that chemicals tended to preferentially increase, as opposed to decrease, the expression of genes downregulated in both juvenile and Lynx1-/- signatures (binomial tests: *P* = 2.3 × 10^−12^ and *P* < 2.2 × 10^−16^) (Figures 2(a) and 2(b)). We focused our subsequent analysis on 50 chemicals (of a total of 1742) that increased genes whose expression was downregulated in both of the critical period signatures, which was a significant overlap (Fisher's exact test: *P* = 2.2 × 10^−16^, OR = 14.4) (Figure 2(b)). Genes downregulated in the critical period signatures are putative “brakes” on developmental brain plasticity, suggesting that these 50 chemicals may disrupt neurodevelopment by prematurely expressing plasticity-dampening molecules.

### 3.2. Chemicals That Dysregulate Critical Period Signatures Converge on Pathogen Response Inflammatory Pathways

**Figure 3:**
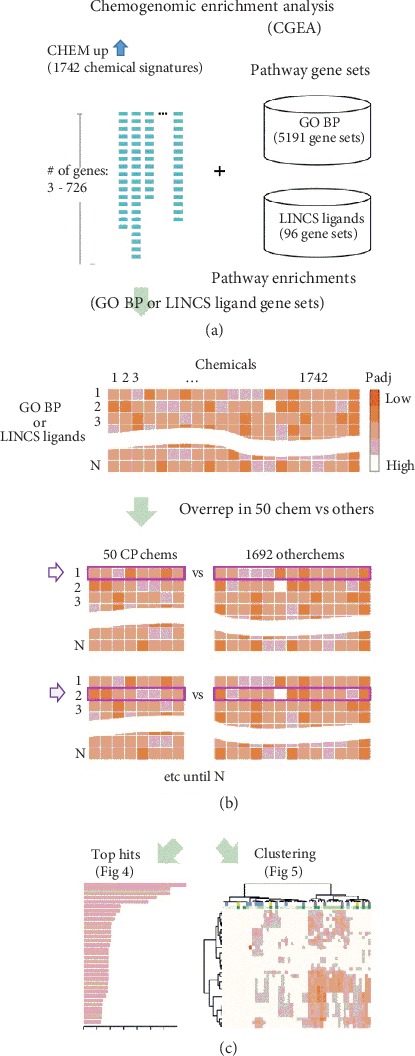
Chemogenomic enrichment analysis (CGEA) workflow. (a) Enrichments of 5191 Gene Ontology (GO) Biological Process (BP) and 96 Library of Integrated Network-based Cellular Signatures (LINCS) ligand gene sets were calculated for 1742 CHEM up signatures. (b) We calculated overrepresentation of pathways in each of 50 chemical signatures that impact critical period signatures, relative to the remaining 1692 chemical signatures. (c) Top overrepresentation hits were calculated (Figure 4), and hierarchical clustering was performed on enrichment Padj values (Figure 5).

**Figure 4:**
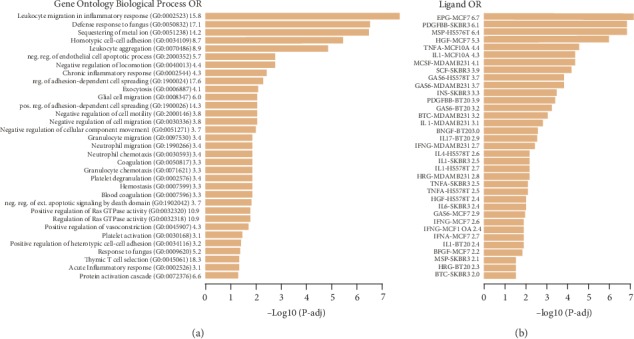
Chemogenomic enrichment analysis of 50 chemicals that increase expression of genes downregulated in the critical period signatures reveals inflammatory, response to pathogen, and immune cell chemotaxis pathways. We computed gene set enrichments for the CHEM up library (1742 chemical signatures) across 5191 Gene Ontology (GO) Biological Process (BP) gene sets and 96 LINCS ligand gene sets to yield 9,042,722 and 167,232 enrichment *P* values, which were corrected for multiple testing using the Benjamini and Hochberg approach. For each biological process or ligand, we calculated the overrepresentation of that gene set (if it was significant after multiple test correction) among the 50 chemicals identified as impacting both juvenile and Lynx1-/- critical period signatures, in comparison to the remaining 1692 chemicals, using a hypergeometric test (hypergea R package implementation). A pathway was considered associated with a chemical if the enrichment Padj < 0.05, yielding (a) 33 GO BP gene sets and (b) 48 LINCS ligand gene sets.

The 50 chemicals shown by GSEA to impact both juvenile and Lynx1-/- signatures were diverse and included pesticides (e.g., pyridaben), antimicrobials (e.g., bacitracin), metals (e.g., mercury), anesthetics (e.g., halothane), and other compounds or mixtures (e.g., vehicle emissions) (Supplementary Table 1). To gain insight into biological effects that might be shared by these diverse chemicals, we applied chemogenomic enrichment analysis (CGEA) by calculating overrepresentation of biological pathways in each of the 50 chemical signatures, relative to the remaining 1692 chemical signatures (see Figure 3 for the workflow). Using 5191 Gene Ontology (GO) Biological Process (BP) gene sets, we identified 33 BPs overrepresented in the 50 chemical signatures (at Padj < 0.05). CGEA enrichments of GO BPs were overwhelmingly associated to response to pathogen, immune cell chemotaxis, and inflammation (Figure 4(a)).

To understand the potential cytokine signaling by which these chemicals induce inflammatory responses, we computed overrepresentations for 96 ligand gene sets derived from the Enrichr library (http://amp.pharm.mssm.edu/Enrichr/) [26] which includes the Library of Integrated Network-based Cellular Signatures (LINCS) database, comprising genes upregulated after exposure to cytokines or growth factors. Consistent with the GO overrepresentations, we observed consistent overrepresentations of genes increased by IL-1 (5/7 IL-1 gene sets) and TNF-*α* (5/5 TNF-*α* gene sets), suggesting that these chemicals mimic an immune response to pathogen at the level of cytokine signaling (Figure 4(b)).

**Figure 5:**
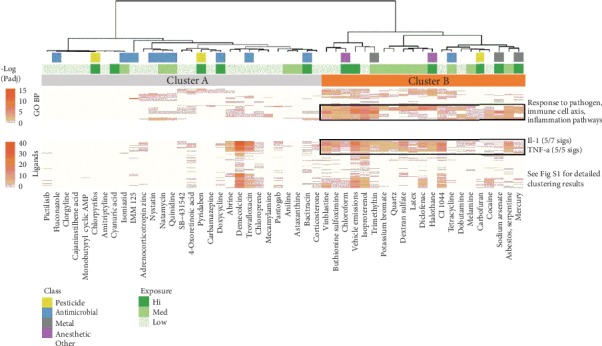
Clustering of chemical pathway enrichments identifies antimicrobial and inflammatory clusters. Hierarchical clustering (Ward D method) on the negative log Padj values of Gene Ontology (GO) Biological Process (BP) and LINCS ligand enrichment analysis revealed two clusters of chemicals. Cluster A (29 chemicals) contains few inflammatory pathway enrichments and 9 of the 10 antimicrobials in the set of 50 chemicals examined, whereas Cluster B contains the majority of enrichments for response to pathogen, inflammation, immune cell chemotaxis, and IL-1/TNF-*α*. See Supplementary Figure 1 for detailed enrichment information.

To determine whether these enrichments were consistent across all 50 chemicals, we performed hierarchical clustering on the negative log Padj values of the BP and ligand enrichments. This analysis yielded two primary clusters: Cluster A (29 chemicals) with few enrichments for inflammatory pathways and instead enriched in antimicrobials and Cluster B (21 chemicals) enriched in inflammatory pathways and more likely to be exposed to humans (Cluster B has 15/21 chemicals with medium or high exposure likelihood versus 10/29 chemicals in Cluster A; 2.07-fold enrichment; binomial test expecting equal likelihood: *P* = 0.103) (Figure 5 and Supplementary Table 1). These results suggest that 50 chemicals that dysregulate critical period signatures segregate into two major clusters; the members of one of these clusters are more likely to be exposed to humans and mimic an immune response to pathogen.

### 3.3. Chemicals That Dysregulate Critical Period Signatures Mimic Lipopolysaccharide-Activated Microglia

Given that CGEA identified enrichments of response to pathogen, immune cell chemotaxis, and inflammatory pathways, including the IL-1 and TNF-*α* pathways, we sought to determine whether these chemicals induce a peripheral pathogen-like inflammatory response in microglia. Microglia, the resident immune cells of the brain, not only survey the landscape for pathogens and cellular detritus but also support neural function and are required for critical period plasticity [27].

**Figure 6:**
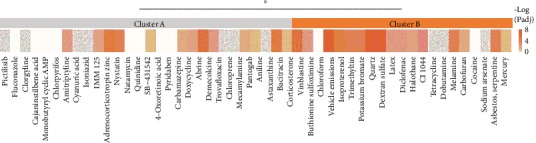
Fifty chemicals mimic the gene expression phenotype induced by LPS-activated microglia. We used Fisher's exact test to calculate the overlap of microglia genes increased by LPS activation to the genes in a given CHEM up signature. 58% of all chemicals were enriched (at Padj < 0.05), and Cluster B was more likely than Cluster A to display this phenotype (Fisher's exact test: OR = 3.8, ^∗^*P* = 0.26). Chemicals ordered as in Figure 5.

We hypothesized that these chemicals activate microglia, shifting them from the “resting-state” phenotype necessary to facilitate plasticity to a vigilant, activated state. To test this hypothesis, we generated a transcriptional signature of lipopolysaccharide- (LPS-) activated microglia, comprising 72 genes increased by LPS [21]. We then assessed this signature for overlap with the genes in a given CHEM up signature. The majority of chemicals (58%) mimicked an activated microglia phenotype at the transcriptional level, and Cluster B was more likely than Cluster A to display this phenotype (Fisher's exact test: OR = 3.8, ^∗^*P* = 0.26) (Figure 6(a) and Supplementary Table 2), indicating that a subset of these chemicals activates microglia in a similar manner to LPS. These analyses indicate that a subset of chemicals that increase the expression of putative brakes on critical period plasticity, and whose gene expression signatures are enriched for inflammatory pathways, induces a transcriptional response similar to that of microglial activation, suggesting that exposure of these environmental chemicals during the critical period could activate microglia, shifting them from their physiological role in plasticity to a state of active watchfulness and disrupting critical period plasticity.

## 4. Discussion

Building on our recent proof-of-principle study [15], we established a transcriptome-based integrative bioinformatics approach to systematically identify environmental chemicals that dysregulate transcriptional signatures of critical periods of cortical plasticity. Previous high-throughput approaches typically used biochemical and cell-based experimental assays focused on a limited number of gene/protein expression or enzymatic activities. Although these assays may themselves be straightforward, they do not necessarily reflect more complex in vivo neurodevelopmental events. On the other hand, in vivo animal assays are low-throughput and only appropriate for the validation of screening results. Due to these limitations, no previous studies have attempted to systematically identify environmental chemicals that disrupt complex in vivo phenomenon such as critical periods of plasticity. Here, leveraging the utility of transcriptional signature matching to identify functional and mechanistic relationships [28], we matched multiple signatures of in vivo critical period plasticity to thousands of chemical signatures derived from public transcriptional data to systematically identify novel childhood critical period toxicant candidates.

The developmental consequences of disruption by these chemicals could be far-reaching. Disruption of the critical period for visual cortex plasticity prevents the development of an important visual function termed binocular matching of orientation preference [29], resulting in a disordered visual experience. Moreover, due to the hierarchical dependency of multiple critical periods (i.e., hearing, vision, language, and cognitive processes) across development, disruption of a sensory-specific critical period might ultimately interfere with higher-order cognitive functions [12]. In addition, given the fact that the mechanisms of plasticity identified in the visual critical period have been generalized to other brain regions and functions [30–32], critical period toxicants identified using the visual model may disrupt plasticity and neurodevelopment in other brain regions and for other functions.

Included among the 50 plasticity-disrupting chemical candidates we identified were both known and novel neurotoxicants with high exposure likelihood including inorganic metals (mercury, sodium arsenate), pesticides (pyridaben, chlorpyrifos, and carbofuran), anesthetics (chloroform and halothane), antimicrobials (bacitracin+nine others), and other chemicals (vehicle emissions, cyanuric acid—a common swimming pool water additive). There is evidence consistent with the ability of these chemicals to disrupt critical periods. For example, mercury levels have increased by 3-fold over the past 100 years, in large part due to power plant emissions and industrial byproducts [33]. Human exposure is primarily through the microorganism-processed methylated form (MeHg), which is found in aquatic organisms consumed as food, such as fish. Perinatal treatment of MeHg to mouse dams (embryonic day 7 (E7) to P7) at a dose of 0.59 mg/kg/day suggested a potential decrease in the maturation of parvalbumin-expressing neurons in the hippocampus of juvenile animals [34], suggesting that MeHg could delay the opening of critical periods, which requires the normal maturation of inhibitory neurons, such as parvalbumin-expressing cells [35]. Moreover, mercury, arsenic, chlorpyrifos, pyridaben, and vehicle emissions have been implicated in the neurodevelopmental disorder autism [20, 36–39], for which the critical period is emerging as a potential period of risk [15].

A large portion of critical period-disrupting candidates were antimicrobials (10 of 50) indicating that the downstream pathways of antimicrobials may ultimately impact brain development. Bacitracin is used in humans as an antibiotic as well as in commercial farming practices to control microbes and in the feed of swine, chickens, and other livestock to promote growth [40]. Given the widespread administration of antibiotics to livestock for human consumption, there is considerable concern about the impact of residual antibiotic in animal products and its impact on human health [41]. Moreover, there is a growing recognition of the importance of the microbiome-immune-neural axis on health and disease and antibiotics can profoundly disrupt healthy microbiomes [42]. Bacitracin disrupts the microbiome and impacts BDNF levels [43], a growth factor involved in the opening of the visual critical period [35].

Given the diversity of the 50 candidate plasticity disruptors, we applied a chemogenomic enrichment analysis (CGEA) approach to identify shared pathways among these chemicals, which included response to pathogen, immune cell chemotaxis, and inflammatory pathways including IL-1 and TNF-*α* cytokine signaling. This suggests that chemicals that disrupt critical period plasticity may be perceived as invaders by the immune system, leading to induction of an inflammatory response. In the brain, this may involve activation of microglia. Should this occur during the critical period, it might shift microglia away from their physiological role in experience-dependent critical period plasticity [27] to a state of active watchfulness in which they are not able to facilitate experience-dependent brain development. Upon exposure to toxicants such as ozone and acetaminophen, peripheral immune cells (e.g., macrophages) activate and induce an inflammatory response that includes cytokines such as TNF, mimicking the response to Gram-negative bacterial pathogens [44]. Given the role of TNF in activating microglia [45–47], soluble transport of TNF across the blood-brain barrier [48] from peripherally stimulated immune cells could activate the stimulus. Future studies should assess the ability of individual chemicals to activate microglia and disrupt critical period plasticity.

This study was limited by the quality and breadth of available chemical data, and a future work will benefit from the toxicology in the 21st Century (Tox21) program, an ongoing effort to systematically profile the effect of tens of thousands of chemicals on the expression of 1500 genes in cell lines [49]. The chemical data used here were derived from heterogeneous tissues in multiple animal and cell models, not specifically focused on neurons or the brain [50]. Hence, specificity for neuronal phenotypes could be improved by extending current efforts to screen for damaging effects of toxicants in human cell lines [51] to neurons derived from human-induced pluripotent stem cells (iPSCs). Finally, we limited ourselves to two models of critical period plasticity; additional models, such as voluntary exercise-induced plasticity [52], may reveal additional insight regarding the mechanisms that can disrupt critical period plasticity.

In summary, we established an integrative bioinformatics paradigm for generating rational hypotheses about the impact of environmental chemicals on critical periods of brain plasticity, as well as their underlying mechanisms, with the goal of identifying targets for therapeutic intervention. This approach could be generalized to other brain phenotypes, allowing systematic assessment of the impact of chemicals on a wide array of brain development phenotypes.

## Data Availability

The data used to support the findings of this study are included within the article.

## Supplementary Materials

Supplementary Materials Supplementary Table 1: chemicals that commonly increase putative brakes of both juvenile and Lynx1-KO transcriptome signatures. Supplementary Table 2: enrichment statistics for fifty chemicals that mimic the gene expression phenotype induced by LPS-activated microglia (sorted by an ascending *P* value). Supplementary Figure 1: expanded results of hierarchical clustering of GO BP and LINCS ligand CGEA for each of the 50 plasticity-disrupting candidate compounds (related to Figure 5).
