# Supplementary material for: Systematic Analysis of Environmental Chemicals That Dysregulate Critical Period Plasticity-Related Gene Expression Reveals Common Pathways That Mimic Immune Response to Pathogen
Source: Neural Plast. 2020 May 5;2020:1673897. doi: 10.1155/2020/1673897 (PMC7222500; doi:10.1155/2020/1673897)

**SUPPLEMENTAL MATERIALS**

**Table Legends:**

**Supplementary Table 1. Chemicals that commonly increase putative brakes of both juvenile and Lynx1-KO transcriptome signatures.** The 50 chemicals shown by GSEA to impact both juvenile and Lynx1-/- signatures were diverse and included pesticides (e.g. pyridaben), antimicrobials (e.g. bacitracine), metals (e.g. mercury), anesthetics (e.g. halothane), and other compounds or mixtures (e.g. vehicle emissions)

**Supplementary Table 2. Enrichment statistics for fifty chemicals that mimic the gene expression phenotype induced by LPS-activated microglia (sorted by ascending P value).** A transcriptional signature of lipopolysaccharide (LPS)-activated microglia, comprising 72 genes increased by LPS (Bennett et al., 2016) were assessed for overlap with the genes in a given CHEM up signature. The majority of chemicals (58%) mimicked an activated microglia phenotype at the transcriptional level and Cluster B were more likely than Cluster A to display this phenotype (Fisher Exact test: OR = 3.8, *P = 0.26).

**Figure Legend:**

**Supplementary Figure 1. Expanded results of hierarchical clustering of GO BP and LINCS Ligand CGEA for each of the 50 plasticity-disrupting candidate compounds (related to Fig 5).** Hierarchical clustering on the negative log Padj values of Gene Ontology (GO) Biological Process (BP) and LINCS ligand enrichment analysis revealed two clusters of chemicals. Cluster A (29 chemicals) contains few inflammatory pathway enrichments and 9 of the 10 antimicrobials in the set of 50 chemicals examined, whereas Cluster B contains the majority of enrichments for response to pathogen, inflammation, immune cell chemotaxis, and IL1/TNFα.

### Supplementary Tables

Supplementary Table 1. Chemicals that commonly increase putative brakes of both juvenile and

Lynx1-KO transcriptome signatures

|  |  |  | Juvenile | | | | | *Lynx1*-KO | | | |  |
| --- | --- | --- | --- | --- | --- | --- | --- | --- | --- | --- | --- | --- |
| Chemical | Classification  - simple | Exposure  likelihood | | Enrichment Score | P-value | P-adj | Enrichment Score | P-value | P-adj | |  |  |
| 4-oxoretinoic acid | Other | Low | | -0.56 | 4.1E-03 | 1.3E-01 | -0.76 | 0.0E+00 | | 2.3E-01 | | |
| Abrine | Other | Low | | -0.61 | 0.0E+00 | 4.4E-03 | -0.57 | 8.7E-03 | | 2.0E-02 | | |
| Adrenocorticotropin zinc | Other | Low | | -0.55 | 1.1E-02 | 1.3E-01 | -0.54 | 4.1E-02 | | 1.6E-01 | | |
| Amitriptyline | Other | Low | | -0.55 | 4.4E-03 | 1.5E-01 | -0.58 | 2.0E-02 | | 4.8E-03 | | |
| Aniline | Other | Med | | -0.68 | 0.0E+00 | 1.6E-02 | -0.60 | 2.7E-02 | | 6.8E-02 | | |
| Asbestos, serpentine | Other | Med | | -0.65 | 0.0E+00 | 3.6E-02 | -0.68 | 7.9E-03 | | 1.7E-01 | | |
| Astaxanthine | Other | Med | | -0.65 | 0.0E+00 | 2.0E-02 | -0.55 | 3.5E-02 | | 1.6E-01 | | |
| Bacitracin | Antimicrobial | Hi | | -0.63 | 1.0E-02 | 6.7E-02 | -0.78 | 0.0E+00 | | 1.6E-01 | | |
| Buthionine sulfoximine | Other | Low | | -0.49 | 0.0E+00 | 5.1E-02 | -0.63 | 0.0E+00 | | 5.3E-02 | | |
| Cajaninstilbene acid | Other | Low | | -0.62 | 1.9E-02 | 1.8E-01 | -0.78 | 2.0E-03 | | 1.7E-01 | | |
| Carbamazepine | Other | Low | | -0.59 | 0.0E+00 | 1.8E-02 | -0.50 | 4.6E-02 | | 8.5E-03 | | |
| Carbofuran | Pesticide | Hi | | -0.65 | 4.4E-03 | 8.1E-02 | -0.72 | 6.4E-03 | | 5.2E-03 | | |
| Chloroform | Anesthetic | Hi | | -0.58 | 8.5E-03 | 8.3E-02 | -0.63 | 4.0E-03 | | 2.4E-01 | | |
| Chloroprene | Other | Low | | -0.46 | 0.0E+00 | 6.5E-03 | -0.39 | 0.0E+00 | | 5.5E-02 | | |
| Chlorpyrifos | Pesticide | Hi | | -0.47 | 2.1E-03 | 1.9E-01 | -0.59 | 1.8E-03 | | 9.5E-02 | | |
| Ci 1044 | Other | Low | | -0.59 | 0.0E+00 | 1.6E-02 | -0.69 | 0.0E+00 | | 1.3E-01 | | |
| Clorgyline | Other | Low | | -0.49 | 0.0E+00 | 2.5E-02 | -0.45 | 5.3E-03 | | 6.2E-03 | | |
| Cocaine | Other | Low | | -0.40 | 0.0E+00 | 8.9E-02 | -0.45 | 0.0E+00 | | 1.5E-01 | | |
| Corticosterone | Other | Low | | -0.44 | 6.2E-03 | 1.9E-01 | -0.46 | 2.8E-02 | | 5.8E-02 | | |
| Cyanuric acid | Other | Hi | | -0.97 | 0.0E+00 | 1.6E-01 | -0.98 | 0.0E+00 | | 1.8E-01 | | |
| Demecolcine | Other | Low | | -0.35 | 0.0E+00 | 2.5E-01 | -0.37 | 0.0E+00 | | 1.3E-01 | | |
| Dextran sulfate | Other | Med | | -0.62 | 0.0E+00 | 1.8E-02 | -0.70 | 0.0E+00 | | 2.3E-01 | | |
| Diclofenac | Other | Med | | -0.56 | 2.3E-03 | 3.8E-02 | -0.71 | 0.0E+00 | | 4.8E-03 | | |
| Dobutamine | Other | Low | | -0.87 | 2.1E-02 | 2.2E-01 | -0.86 | 2.6E-02 | | 5.8E-03 | | |
| Doxycycline | Antimicrobial | Hi | | -0.73 | 0.0E+00 | 2.0E-02 | -0.76 | 3.7E-03 | | 1.8E-01 | | |
| Fluconazole | Antimicrobial | Low | | -0.92 | 0.0E+00 | 5.0E-02 | -0.86 | 1.2E-02 | | 3.0E-02 | | |
| Halothane | Anesthetic | Hi | | -0.96 | 0.0E+00 | 3.0E-02 | -0.95 | 0.0E+00 | | 1.5E-01 | | |
| Imm 125 | Antimicrobial | Low | | -0.84 | 1.0E-02 | 1.3E-01 | -0.87 | 2.0E-02 | | 7.1E-02 | | |
| Isoniazid | Antimicrobial | Med | | -0.78 | 8.7E-03 | 9.5E-02 | -0.86 | 0.0E+00 | | 1.4E-01 | | |
| Isoproterenol | Other | Low | | -0.35 | 0.0E+00 | 1.9E-01 | -0.38 | 0.0E+00 | | 3.8E-02 | | |
| Latex | Other | Med | | -0.52 | 2.1E-03 | 2.0E-01 | -0.53 | 2.9E-02 | | 1.6E-01 | | |
| Mecamylamine | Other | Low | | -0.77 | 6.3E-03 | 9.4E-02 | -0.81 | 1.3E-02 | | 1.8E-01 | | |
| Melamine | Other | Med | | -0.79 | 3.8E-02 | 1.9E-01 | -0.87 | 6.3E-03 | | 1.0E-01 | | |
| Mercury | Metal | Hi | | -0.43 | 0.0E+00 | 9.6E-02 | -0.44 | 1.7E-03 | | 9.4E-02 | | |
| Monobutyryl cyclic amp | Other | Low | | -0.91 | 2.2E-03 | 9.7E-02 | -0.93 | 6.3E-03 | | 1.5E-01 | | |
| Natamycin | Antimicrobial | Med | | -0.92 | 0.0E+00 | 1.3E-01 | -0.96 | 0.0E+00 | | 1.1E-01 | | |
| Nystatin | Antimicrobial | Low | | -0.87 | 6.2E-03 | 1.3E-01 | -0.93 | 2.1E-03 | | 1.2E-01 | | |
| Pantogab | Other | Low | | -0.40 | 0.0E+00 | 9.2E-02 | -0.41 | 3.2E-03 | | 8.1E-02 | | |
| Pictilisib | Other | Low | | -0.48 | 0.0E+00 | 1.7E-02 | -0.39 | 6.8E-03 | | 2.9E-02 | | |
| Potassium bromate | Other | Med | | -0.77 | 2.1E-03 | 3.7E-02 | -0.77 | 1.7E-02 | | 1.8E-01 | | |
| Pyridaben | Pesticide | Hi | | -0.72 | 0.0E+00 | 5.4E-03 | -0.68 | 1.9E-03 | | 7.0E-02 | | |
| Quartz | Other | Med | | -0.72 | 2.1E-03 | 1.8E-02 | -0.77 | 4.1E-03 | | 3.9E-02 | | |
| Quinidine | Antimicrobial | Med | | -0.83 | 1.3E-02 | 1.1E-01 | -0.89 | 2.1E-03 | | 1.9E-02 | | |
| Sb-431542 | Other | Low | | -0.84 | 4.5E-03 | 1.1E-01 | -0.80 | 2.7E-02 | | 9.5E-02 | | |
| Sodium arsenate | Metal | Hi | | -0.39 | 0.0E+00 | 2.5E-02 | -0.39 | 0.0E+00 | | 1.1E-01 | | |
| Tetracycline | Antimicrobial | Med | | -0.49 | 0.0E+00 | 2.3E-02 | -0.44 | 3.6E-03 | | 1.6E-01 | | |
| Trimethyltin | Metal | Med | | -0.51 | 2.1E-02 | 1.9E-01 | -0.69 | 0.0E+00 | | 1.7E-02 | | |
| Trovafloxacin | Antimicrobial | Low | | -0.41 | 4.4E-03 | 2.0E-01 | -0.41 | 2.7E-02 | | 2.5E-01 | | |
| Vehicle emissions | Other | Hi | | -0.39 | 0.0E+00 | 1.9E-01 | -0.47 | 0.0E+00 | | 9.4E-02 | | |
| Vinblastine | Other | Low | | -0.53 | 2.1E-02 | 2.0E-01 | -0.73 | 0.0E+00 | | 1.3E-02 | | |

Supplementary Table 2. Enrichment statistics for fifty chemicals that mimic the gene expression phenotype induced by LPS-activated microglia (sorted by ascending *P* value)

| Chemical | Universe Size | Gene Set Size | Total Hits | Expected Hits | Observed Hits | P-value | P-adj | OR |
| --- | --- | --- | --- | --- | --- | --- | --- | --- |
| Dextran Sulfate | 15471 | 91 | 74 | 0.435265982 | 10 | 1.57558E-11 | 7.87791E-10 | 22.974 |
| Quartz | 15471 | 33 | 74 | 0.157843708 | 7 | 1.65696E-10 | 2.7616E-09 | 44.348 |
| Vehicle Emissions | 15471 | 246 | 74 | 1.176653093 | 13 | 1.33979E-10 | 2.7616E-09 | 11.048 |
| Demecolcine | 15471 | 456 | 74 | 2.18111305 | 14 | 2.7721E-08 | 3.28712E-07 | 6.419 |
| Vinblastine | 15471 | 40 | 74 | 0.191325706 | 6 | 3.28712E-08 | 3.28712E-07 | 31.36 |
| Bacitracin | 15471 | 25 | 74 | 0.119578566 | 5 | 1.0763E-07 | 8.96921E-07 | 41.814 |
| Nystatin | 15471 | 12 | 74 | 0.057397712 | 4 | 2.31872E-07 | 1.65623E-06 | 69.689 |
| Isoproterenol | 15471 | 595 | 74 | 2.845969879 | 14 | 7.19847E-07 | 4.49904E-06 | 4.919 |
| Asbestos, Serpentine | 15471 | 50 | 74 | 0.239157133 | 5 | 3.91115E-06 | 1.95558E-05 | 20.907 |
| Trimethyltin | 15471 | 49 | 74 | 0.23437399 | 5 | 3.53314E-06 | 1.95558E-05 | 21.333 |
| Natamycin | 15471 | 8 | 74 | 0.038265141 | 3 | 5.78254E-06 | 2.62843E-05 | 78.4 |
| Potassium Bromate | 15471 | 26 | 74 | 0.124361709 | 4 | 6.65674E-06 | 2.77364E-05 | 32.164 |
| Melamine | 15471 | 10 | 74 | 0.047831427 | 3 | 1.23061E-05 | 4.7331E-05 | 62.72 |
| CI 1044 | 15471 | 67 | 74 | 0.320470558 | 5 | 1.67368E-05 | 5.97744E-05 | 15.602 |
| Abrine | 15471 | 136 | 74 | 0.650507401 | 6 | 4.68716E-05 | 0.000156239 | 9.224 |
| Cyanuric Acid | 15471 | 4 | 74 | 0.019132571 | 2 | 0.000134585 | 0.00042058 | 104.534 |
| Buthionine Sulfoximine | 15471 | 105 | 74 | 0.502229979 | 5 | 0.000145347 | 0.000427492 | 9.956 |
| Diclofenac | 15471 | 63 | 74 | 0.301337987 | 4 | 0.000232046 | 0.000644571 | 13.274 |
| Trovafloxacin | 15471 | 122 | 74 | 0.583543404 | 5 | 0.000292989 | 0.000771023 | 8.568 |
| Latex | 15471 | 127 | 74 | 0.607459117 | 5 | 0.000352756 | 0.000881889 | 8.231 |
| Adrenocorticotropin zinc | 15471 | 47 | 74 | 0.224807705 | 3 | 0.001464514 | 0.00332844 | 13.345 |
| Halothane | 15471 | 12 | 74 | 0.057397712 | 2 | 0.001444154 | 0.00332844 | 34.845 |
| Doxycycline | 15471 | 24 | 74 | 0.114795424 | 2 | 0.005819271 | 0.012650589 | 17.422 |
| Carbofuran | 15471 | 26 | 74 | 0.124361709 | 2 | 0.006810271 | 0.014188064 | 16.082 |
| Pantogab | 15471 | 254 | 74 | 1.214918234 | 5 | 0.007361143 | 0.014722287 | 4.116 |
| Aniline | 15471 | 30 | 74 | 0.14349428 | 2 | 0.009003658 | 0.017314727 | 13.938 |
| Corticosterone | 15471 | 103 | 74 | 0.492663693 | 3 | 0.013201976 | 0.024448103 | 6.089 |
| Mercury | 15471 | 194 | 74 | 0.927929675 | 4 | 0.013946656 | 0.024904744 | 4.311 |
| 4-oxoretinoic Acid | 15471 | 45 | 74 | 0.215241419 | 2 | 0.019568842 | 0.033739383 | 9.292 |
| Chloroprene | 15471 | 680 | 74 | 3.252537005 | 7 | 0.043583883 | 0.072639806 | 2.152 |
| Fluconazole | 15471 | 10 | 74 | 0.047831427 | 1 | 0.046828249 | 0.075529434 | 20.907 |
| IMM 125 | 15471 | 12 | 74 | 0.057397712 | 1 | 0.055930918 | 0.087392059 | 17.422 |
| Dobutamine | 15471 | 13 | 74 | 0.062180855 | 1 | 0.06045004 | 0.09159097 | 16.082 |
| Mecamylamine | 15471 | 15 | 74 | 0.07174714 | 1 | 0.06942436 | 0.102094647 | 13.938 |
| Isoniazid | 15471 | 16 | 74 | 0.076530282 | 1 | 0.073879756 | 0.105542508 | 13.067 |
| Cajaninstilbene Acid | 15471 | 28 | 74 | 0.133927994 | 1 | 0.12573075 | 0.174626042 | 7.467 |
| Sodium Arsenate | 15471 | 743 | 74 | 3.553874992 | 6 | 0.144223195 | 0.194896209 | 1.688 |
| Astaxanthine | 15471 | 37 | 74 | 0.176976278 | 1 | 0.16272952 | 0.214117789 | 5.65 |
| Tetracycline | 15471 | 167 | 74 | 0.798784823 | 2 | 0.190188819 | 0.24383182 | 2.504 |
| Pyridaben | 15471 | 51 | 74 | 0.243940275 | 1 | 0.217234794 | 0.271543493 | 4.099 |
| Cocaine | 15471 | 316 | 74 | 1.511473079 | 2 | 0.448162653 | 0.546539821 | 1.323 |
| Clorgyline | 15471 | 140 | 74 | 0.669639972 | 1 | 0.490480723 | 0.583905623 | 1.493 |
| Pictilisib | 15471 | 212 | 74 | 1.014026243 | 0 | 1 | 1 | 0 |
| SB-431542 | 15471 | 18 | 74 | 0.086096568 | 0 | 1 | 1 | 0 |
| Amitriptyline | 15471 | 49 | 74 | 0.23437399 | 0 | 1 | 1 | 0 |
| Carbamazepine | 15471 | 81 | 74 | 0.387434555 | 0 | 1 | 1 | 0 |
| Chloroform | 15471 | 47 | 74 | 0.224807705 | 0 | 1 | 1 | 0 |
| Monobutyryl cyclic AMP | 15471 | 5 | 74 | 0.023915713 | 0 | 1 | 1 | 0 |
| Chlorpyrifos | 15471 | 59 | 74 | 0.282205417 | 0 | 1 | 1 | 0 |
| Quinidine | 15471 | 13 | 74 | 0.062180855 | 0 | 1 | 1 | 0 |

### Supplementary Figure

Supplementary Figure 1. Expanded results of hierarchical clustering of GO BP and LINCS Ligand CGEA for each of the 50 plasticity-disrupting candidate compounds (related to Fig 5).


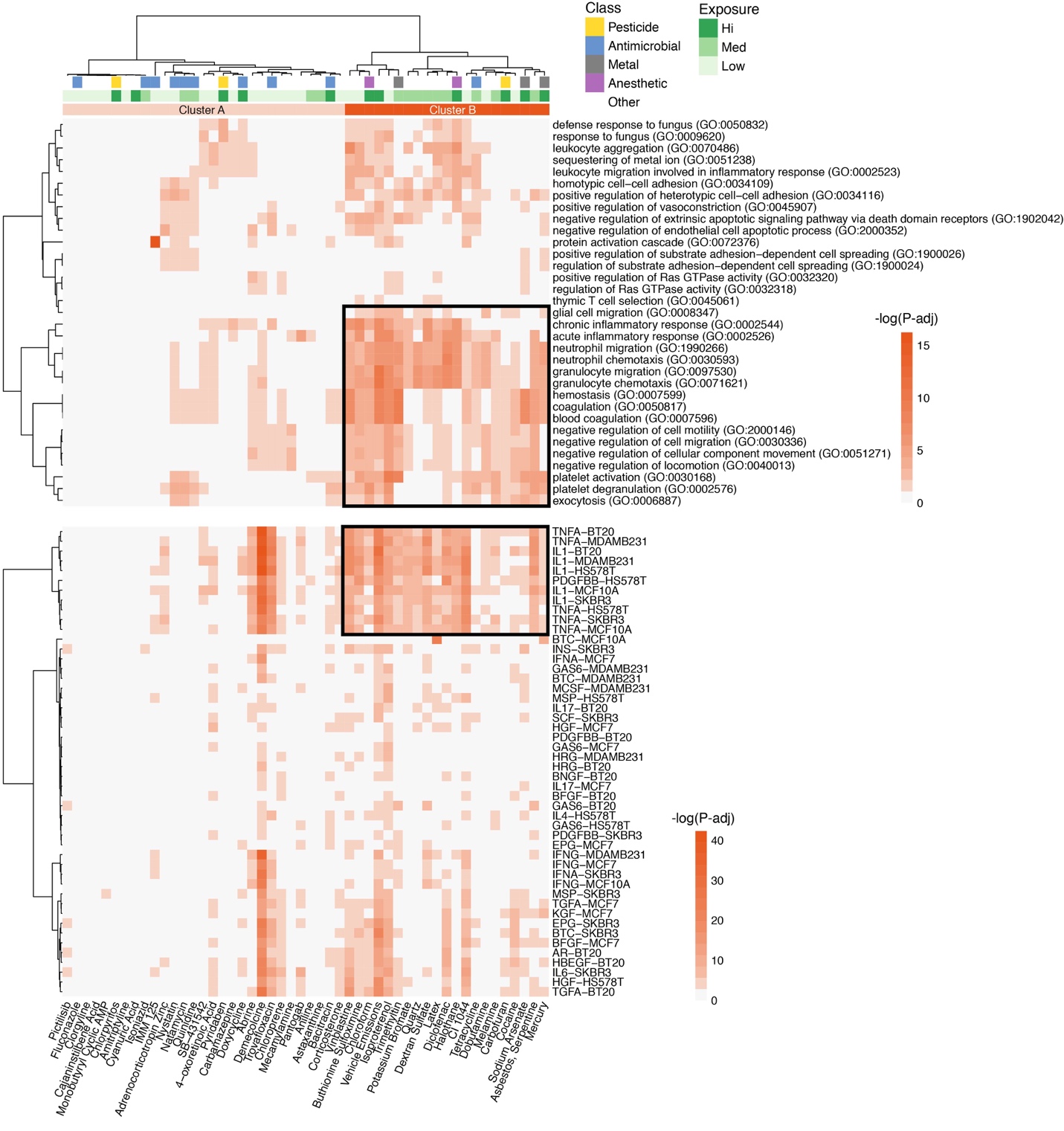

Supplement: Supplementary Materials — Supplementary Table 1: chemicals that commonly increase putative brakes of both juvenile and Lynx1-KO transcriptome signatures. Supplementary Table 2: enrichment statistics for fifty chemicals that mimic the gene expression phenotype induced by LPS-activated microglia (sorted by an ascending P value). Supplementary Figure 1: expanded results of hierarchical clustering of GO BP and LINCS ligand CGEA for each of the 50 plasticity-disrupting candidate compounds (related to Figure 5). [file 1673897.f1.docx]
